# Genomics and multiomics in the age of precision medicine

**DOI:** 10.1038/s41390-025-04021-0

**Published:** 2025-04-04

**Authors:** Srinivasan Mani, Seema R. Lalani, Mohan Pammi

**Affiliations:** 1 Department of Pediatrics, University at Buffalo, Buffalo, NY USA; 2 Department of Molecular and Human Genetics, Baylor College of Medicine, Houston, TX USA; 3 Division of Neonatology, Department of Pediatrics, Texas Children’s Hospital, Houston, TX USA

## Abstract

**Abstract:**

Precision medicine is a transformative healthcare model that utilizes an understanding of a person’s genome, environment, lifestyle, and interplay to deliver customized healthcare. Precision medicine has the potential to improve the health and productivity of the population, enhance patient trust and satisfaction in healthcare, and accrue health cost-benefits both at an individual and population level. Through faster and cost-effective genomics data, next-generation sequencing has provided us the impetus to understand the nuances of complex interactions between genes, diet, and lifestyle that are heterogeneous across the population. The emergence of multiomics technologies, including transcriptomics, proteomics, epigenomics, metabolomics, and microbiomics, has enhanced the knowledge necessary for maximizing the applicability of genomics data for better health outcomes. Integrative multiomics, the combination of multiple ‘omics’ data layered over each other, including the interconnections and interactions between them, helps us understand human health and disease better than any of them separately. Integration of these multiomics data is possible today with the phenomenal advancements in bioinformatics, data sciences, and artificial intelligence. Our review presents a broad perspective on the utility and feasibility of a genomics-first approach layered with other omics data, offering a practical model for adopting an integrated multiomics approach in pediatric health care and research.

**Impact:**

- Precision medicine provides a paradigm shift from a conventional, reactive disease control approach to proactive disease prevention and health preservation.
- Phenomenal advancements in bioinformatics, data sciences, and artificial intelligence have made integrative multiomics feasible and help us understand human health and disease better than any of them separately.
- The genotype-first approach or reverse phenotyping has the potential to overcome the limitations of the phenotype-first approach by identifying new genotype-phenotype associations, enhancing the subclassification of diseases by widening the phenotypic spectrum of genetic variants, and understanding functional mechanisms of genetic variations.

## Introduction

Precision medicine, also known as personalized or individualized medicine, is a novel healthcare approach that utilizes the understanding of a person’s genome, environment, lifestyle, and interplay to deliver customized healthcare choices for prevention, diagnosis, and treatment.^1^ Precision medicine provides a paradigm shift from a conventional, reactive disease control approach to proactive disease prevention and health preservation.^2^ We can understand an individual’s short—and long-term disease risks at a molecular level, make predictions regarding health trajectories, and implement preventive measures tailored to the individual, considering their environmental influences and genomic profile.^3^ We can develop biomarkers for early detection of diseases, monitor their progression, and develop novel targeted therapies that could interrupt disease progression and restore health. We may be able to select therapeutics best suited to an individual’s molecular profile, maximizing efficacy and limiting adverse effects. The healthcare transformation based on the precision medicine approach has the potential to provide rich dividends for the upfront costs involved in establishing the infrastructure.^4^ Precision medicine may improve health and productivity of the population, enhance patient trust and satisfaction in healthcare, and accrue health cost-benefits both at an individual and population level.^5–7^

The genomics revolution has laid the foundation for realizing the promise of precision medicine and P4 (predictive, preventive, personalized, and participatory) healthcare.^8^ The Human Genome Project in 2003 helped scientists understand the framework of human biology better and gave them a deeper insight into the etiology of common non-communicable diseases. Through faster and cost-effective genomics data, next-generation sequencing provided the impetus to understand the nuances of complex interactions between genes, diet, and lifestyle that are heterogeneous across the population. The other omics technologies, including transcriptomics, proteomics, epigenomics, metabolomics, and microbiomics, have emerged, enhancing the knowledge necessary for maximizing the applicability of genomics data for better health outcomes. Integration of these multiomics data is possible today with the phenomenal advancements in bioinformatics, data sciences, and artificial intelligence (AI).^9^

**Fig. 1:**
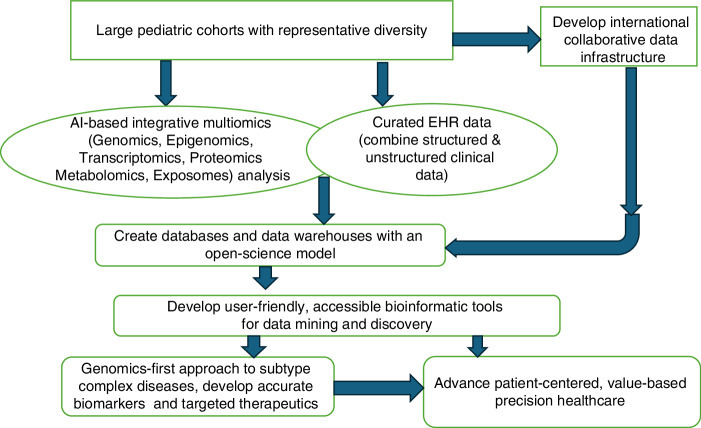
A road map to AI-based integrative multiomics approach for precision medicine in pediatric healthcare and research.

Integrative genomics (with other omics) can help us understand the heterogeneous etiopathogenesis of complex pediatric diseases and create a framework for a precision medicine approach. Breaking down overlapping disease spectrums into definitive subtypes based on an integrative multiomics approach incorporated with clinical data from the EMR can be very valuable and may lead to targeted therapy. Integrated multiomics data of large cohorts with representative population diversity could provide valuable insights into the epidemiology of a disease.^10–12^ Our review is organized into nine broad sections following the roadmap for a practical model for adopting an AI-based integrative multiomics approach that we propose in this review to deliver precision medicine in pediatric health care and research. (Fig. 1) First, we discuss the importance and current state of large-scale longitudinal cohorts, followed by a discussion on innovations and advances in genomics and other omics, and the role of bioinformatics and AI in integrating multiomics. Then, we describe the scope of integrating electronic health records with multiomics data, the current state of genomic databases, data mining technologies, and bioinformatics. Finally, we highlight the clinical and research applications of integrative multiomics, discuss the utility and feasibility of a genomics-first approach with a practical model for adoption, and discuss the challenges and future of precision medicine.

## Longitudinal cohorts

Large prospective cohorts are the backbone of clinical epidemiology. They help us understand the genetic determinants of health and disease, environmental exposures and risk factors, the natural history of a disease, modifiers of disease progression, response to treatment, and long-term prognosis at a population level. Understanding the importance of such longitudinal data in the context of genomics and multiomics, several national public-funded research agencies have invested in developing huge longitudinal cohorts. Table 1 depicts the various longitudinal cohorts available for researchers to access genomic data. Unfortunately, many of the available cohorts have not included children. Developing pediatric cohorts and expanding the recruitment of the pediatric population into the existing cohorts will be crucial in understanding the genetic epidemiology of diseases in children, including infants and newborns.

**Table 1:** Longitudinal Cohorts.

No.	Longitudinal Cohort	Aim	Sample size	Population	Data type	Country
1.	Million Veterans Program	To study the effects of genes, lifestyle, exposures, and military experience on veterans	Greater than 1 million (as of November 2023)	Veterans Age > 18 yrs Race—25% non-white Female—10%	Microarray, WES, WGS, methylation, proteomics, and metabolomics	United States
2.	Gabriella Miller Kids First Pediatric Research Program	To understand the biology of childhood cancers and structural birth defects	Greater than 31000	Age—0–99 yrs Race—11% non-white Female—46%	WES, WGS, RNA-Seq, linked-read WGS, long-read sequencing	United States
3.	‘All of Us’ research program	To collect data from EHR, clinical measurements, digital health technology data, and biospecimens genomic analysis.	Greater than 413,000	Age > 18 yrs Race—49% non-white Sexual or gender minority—13%	WGS, Genetic microarray	United States
4.	UK Biobank	To understand the genetic and non-genetic determinants of health	Greater than 500,000	Age—40–69 yrs Race—14% non-white Female – 38%	WGS, WES, and Whole-genome microarray	United Kingdom
5.	Canadian Partnership for Tomorrow’s Health’	To determine the effect of genes, environment, behavior, and family health history on chronic diseases	307,017	Age—30–74 yrs Race—16% non-white Female—62%	Genotyping, WGS, WES, RNA sequencing, single-cell sequencing	Canada
6.	National Cancer Institute Cohort Consortium	To create an extensive international collaboration to combine a massive quantity of data and biospecimens and enable the conduct of cancer research at a scale and pace not possible before	58 cohorts	Age—mostly adults > 25 yrs Race- predominantly white	Genetic Array WGS, WES, and epigenomic marks	United States, Canada, Europe, Southeast Asia, Mexico, Australia, Iran
7.	International Hundred Thousand Plus Cohort Consortium	To form a consortium of large cohorts and their data linked across them	50 million participants (103 cohorts)		WGS, WES, Genetic array, transcriptomics, proteomics data	43 countries

*WES* whole exome sequencing, *WGS* whole genome sequencing.

Diversity in genomic and multiomics datasets is an essential factor necessary to achieve equity in genomic healthcare and to bring the benefits of precision medicine to the entire population.^13^ It is estimated that participants of European descent constitute 86.3% of all the genomic studies ever conducted worldwide.^14^ The participants of African, South Asian, and Hispanic descent together constitute less than 10% of the studies. In addition to the limited representation in the existing datasets, the applicability of the human reference genome to diverse populations is questionable because of the origin of nearly three-fourths of the reference sequences from a single donor in the US.^15^ Underrepresented populations in genomic research belong to the minority and indigenous groups in the research-intense nations of the developed world and populations from low- and middle-income countries.^14^ Common barriers include lack of a diverse, skilled workforce, funding, trust among the underrepresented population, institutional capacity, and political will. A community-based participatory research framework should include identifying the context of the genomic research relevant to the stakeholders, establishing a diverse cross-sector stakeholder team, creating genomic infrastructure to answer community-centered research questions, collecting data that are culture-sensitive and adaptable to stakeholder feedback, and utilizing the research results to positively impact the health of the community and local health policy.^16^ The existing research funding mechanisms in the Organization of Economic Cooperation and Development (OECD) countries should be capitalized for equitable collaborations and capacity building in resource-limited settings.^17^ The investments in establishing diverse international consortia for genomic research by OECD countries will return mutual benefits.^18^ While aiming to create diverse datasets for genomic research, investigators should understand that race and ethnicity are sociopolitical constructs, and they do not equate to the genetics and ancestry of an individual.^19^

Accurate genomic sequence interpretation is as important as having a representative reference genome for comparison.^20^ There are only 5200 known disease-associated genes, but more than 90,000 variants exist. Only a quarter of these variants have their pathological significance classified, while the rest of them are classified as variants of unknown significance. The classification of a huge number of known sequence variants in the population and storing them in easily accessible databases is important for the accurate interpretation of genomic data. The Genome Aggregation Database (gnomAD) is one of the largest and most widely used public resources for sequencing-based population variation data.^21^ It is particularly valuable as a source of putatively benign variants found in the human population. Among its key features are constraint metrics, which are extensively used to support the interpretation of genes and variants linked to rare diseases. Databases such as ClinVar, the Human Gene Mutation Database (HGMD), OMIM, GeneReviews, the Clinical Genome Resource (ClinGen), DECIPHER, and Mouse Genome Informatics (MGI) are important resources for the clinical interpretation of genetic variants.^22^ To further assist in variant interpretation, various tools have been developed. These include software that complies with ACMG/AMP guidelines and machine learning (ML) models designed to differentiate between pathogenic and benign variants. Prediction tools such as MutationTaster2, PolyPhen, MutationAssessor, CADD, DeepVariant, GATK, REVEL, and phyloP are often employed for analyzing genetic variants. ML-based variant classification and interpretation tools are advantageous compared to statistics-based predictors because they are data-driven and yield probabilistic pathogenicity scores for prioritizing variants of unknown significance.^23^

## Genomics and other omics

The Human Genome Project (HGP) was an ambitious collaborative team effort funded by the US National Institutes of Health involving 20 centers across six countries. It was launched in 1990 and completed in 2003.^24^ This project used the Sanger sequencing method to sequence the open chromatic portion of the human genome and reported a sequence of 2.85 billion nucleotides (approximately 85–90% of the human genome) with minimal interruptions. HGP reported that the human genome contains only 20,000–25,000 protein-coding genes and provided a reference sequence for the future. HGP reshaped the research undertaken to understand human biology, disease states, and their treatment.^25^ It created the need to decipher complex interactions within the human body system at microscopic and macroscopic levels, highlighting the importance of the systems biology approach in biomedical research. HGP spurred the rapid development of technologies necessary for this approach in computation, mathematics, genomic data science, and AI. Another significant contribution of HGP to biomedical research is showcasing the success and potential of team science and open-source data sharing.^26^

The chain termination method developed by Frederick Sanger in 1977 is considered the gold standard for DNA sequencing because of its excellent accuracy in base calling. However, this method yields low throughput because the DNA can be sequenced only in small fragments at a given time. This disadvantage was overcome by newer high throughput technologies that use massively parallel DNA sequencing platforms collectively called next-generation sequencing (NGS).^27,28^ NGS includes various methods like sequencing by synthesis, pyrosequencing, sequencing by ligation, and ion semiconductor sequencing. Sequencing by synthesis using polymerase chain reaction (PCR) is the most widely used method for genome and exome sequencing. Technological refinements aimed at improved accuracy and reduced cost have led to significant advancements in the NGS platforms. For example, the popular HiSeq technology by Illumina had an output capacity in the range of 1.6–1.8 terabytes (Tb), 5.3-6 billion reads per run with a maximum read length of 2 ×150 base pairs (bp) in the year 2014 have advanced to NovaSeq technology that can give an output in the range of 6–16 Tb, 20–52 billion reads per run with maximum read lengths of up to 2 ×250 bp in 2022. There was a significant cost reduction during the same period of technological advancement. The cost of genome sequencing was reduced from 1392 USD in November 2018 to 525 USD in May 2022, per the recent US National Human Genome Research Institute data.^29^ Recently, a newer method of NGS developed by Ultima Genomics utilizing single-end sequencing technology using silicon wafers instead of flow cells has decreased costs to as low as $100 per sample.^30^

Alternative sequencing technology that uses novel methods such as single-molecule real-time sequencing (SMRT) and nanopore sequencing (ONT) without DNA amplification by PCR has been developed in recent years.^31^ The advantages of SMRT and ONT include the ability to sequence long fragment DNA molecules with a read length of 30–50 KB, single-nucleotide resolution, detection of base modifications like methylation patterns used in epigenetic studies, direct RNA sequencing, and proven utility in metagenomics and transcriptomics.^32^ Another cutting-edge sequencing technology on the horizon is single-cell in-situ nucleic acid sequencing. This technology overcomes the loss of spatial information of the transcriptome when cells are separated from the tissue for RNA sequencing.^33,34^

*Transcriptomics*, the study of RNA transcripts including their patterns of splicing, polyadenylation, fusion, isoform quantification and non-translated functional forms, has advanced from microarray to RNA-sequencing (RNA-Seq) technology.^35,36^ RNA-Seq uses massively parallel sequencing to identify the RNA expression at a genome level. RNA-Seq can be classified into bulk RNA-Seq, the study of transcriptional profile of whole tissue, single cell RNA-Seq (scRNA-Seq), study of transcriptional profile at an individual cellular level and spatial RNA-Seq (spRNA-Seq), study of transcriptional profile with a spatial context.^37^ The above-mentioned technological advancements in transcriptomics have impacted our understanding and clinical care of several pediatric diseases. Recognition of gene fusions are vital for risk-based therapies to improve outcomes in children with acute lymphoblastic leukemia.^38^ An Australian study incorporated the RNA-Seq into the routine clinical diagnostic pipeline and found enhanced risk-based classification in a cohort of pediatric ALL patients.^39^ As a part of an ongoing multicenter clinical trial by the Dana Farber Cancer Institute ALL consortium, RNA-Seq was used to study the utility of transcriptomics to augment conventional cancer diagnostics.^40^ The study reported that RNA-Seq identified 56 gene fusions and 141 missense mutations of clinical significance missed by routine tests. A recent study on autism spectrum disorder (ASD) utilized single nucleus RNA sequencing analysis and identified broad transcriptomic dysregulation in the brain specifically the excitatory neurons and glial cells.^41^

The variations in the translated proteins coded by a single gene called the proteoforms, play an essential role in the substantial diversity in the phenotype provided by the limited genes.^42^ Advances in proteomic analysis, such as mass spectrometry and nuclear magnetic resonance mass spectroscopy, allow in-depth analysis of an individual’s proteomic profile, aiming to provide phenotype-genotype correlation that helps us better understand disease pathophysiology and provide new drug targets.^43^ A small pilot study (*n* = 6) evaluated the association of focal segmental glomerulosclerosis (FSGS) with peritoneal membrane fibrosis in children undergoing peritoneal dialysis. The study used machine learning to perform comparative proteomic analysis of mesothelial exosomes in the peritoneal fluid effluent and found 40 distinct proteins to identify FSGS patients with 100% accuracy.^44^ A retrospective observational study aimed to describe the changes in the genome and proteome in pediatric patients with acute lymphoblastic leukemia (ALL) during disease progression.^45^ The study reported the stability of the tumor clones’ genome and proteome during disease progression, demonstrating the benefit of combining proteomics with genomics in the diagnosis of pediatric ALL.

The study of metabolome, molecules smaller than proteins from biological samples, provides real-time insights into phenotypic variations in the disease processes. Small molecule metabolites are increasingly recognized for their role in physiological and pathological processes through cellular signaling mechanism, immune function, and mediating the effects of environmental stimuli.^46^ Metabolomics is an emerging “omics” field that can take us closer to correlating the genotype with the phenotype, including the interplay of envirotype in its analysis.^47^ The technological advancements in mass spectrometry combined with liquid or gas chromatography have taken this field from targeted small-scale analysis of selected metabolites that are tissue-specific to a non-targeted approach that has great potential to subclassify a disease based on variations in metabolic dysfunction, identify novel biomarkers and drug targets.^48,49^ A large prospective metabolomic study (*n* = 708) including children between 18 and 48 months of age with ASD aimed to classify participants based on metabolic alterations, discovered and validated 34 metabotypes, ratios of 39 plasma metabolite pairs related to amino acid and mitochondrial energy metabolisms, that could reliably differentiate children with ASD and typical development.^50^ A cross-sectional study (*n* = 3290 families) comparing the diagnostic rates of untargeted metabolomic profiling with traditional newborn metabolic testing (combination of plasma amino acids, plasma acylcarnitine profile, and urine organic acids) to identify inborn errors of metabolism found that the case finding by untargeted metabolomic approach was approximately 6-fold higher (7.1% vs 1.3%).^51^ A sub-analysis of the Canadian Healthy Infant Longitudinal Development Study (CHILD) cohort (*n* = 647) compared the fecal metabolites analyzed by nuclear magnetic resonance spectroscopy and secretory immunoglobulin A measured at 3–4 months of age, with BMI z scores at 1 and 3 years of age after controlling for environmental factors such as type of delivery, intrapartum antibiotic prophylaxis, birthweight, breastfeeding and solid food introduction.^52^ The study found that fecal formate and butyrate levels in non-exclusively breastfed infants at 3-4 months correlated with the BMI z score at preschool age.

*Epigenomics* systematically studies the reversible, mostly heritable DNA modifications or the proteins attached to the DNA, such as DNA methylation, histone modification, chromatin accessibility, and non-coding RNA that regulate the gene expression without altering the DNA sequence.^53^ Understanding the regulatory component that makes up 98% of our genome is extremely important to make an accurate cell-specific interpretation of the information contained in the genome. Recent technological leaps, including the NGS, have enabled the development of reference epigenomic maps for various cell and tissue types.^54,55^ DNA methylation studies utilize platforms such as whole genome bisulfite sequencing, methylated DNA immunoprecipitation sequencing, and methylation-sensitive restriction enzyme sequencing. Studies looking at histone modifications use chromatin immunoprecipitation assay with sequencing. Investigating chromatin accessibility utilizes DNase cleavage with sequencing, high throughput chromosome conformation capture, transposase-accessible chromatin with sequencing, and chromatin loop reorganization using clustered regularly interspaced short palindromic repeats technology.^56^ Non-coding RNAs of interest in epigenomic studies are long non-coding RNA and micro RNA. They are studied using NGS-based RNA sequencing platforms.^57^ A US birth cohort (*n* = 954) study found that in-utero exposure to maternal diabetes was associated with distinct DNA methylation patterns measured in the cord blood using genome-wide CpG analysis.^58^ The investigators were able to map the methylated CpG sites to specific genes and showed their predictive capacity for preterm birth. An epigenome-wide meta-analysis conducted by the Pregnancy and Childhood consortium, including 17 cohorts with 1299 participants (668 newborns and 631 children), found an association between DNA methylation patterns (9 CpG sites and 35 regions) in the newborn period and risk of school-age asthma.^59^ In children, 179 differentially methylated CpG sites and 36 regions predicted the risk and severity of asthma.

Exposomes are an aggregate of environmental, dietary, behavioral, and microbial exposures in a person’s life combined with the biological responses to such exposures.^60^ The systematic study of exposomes can be considered an extension of metabolomics, including chemicals in the environment and diet and those produced in the body as a response to sleep, regular exercise, or a lack thereof. The emerging field of exposomes holds excellent promise but currently lags behind other omics technologies.^61^ Hu et al. recently proposed a novel workflow to analyze and quantify human exposomes using an untargeted approach using gas chromatography high-resolution mass spectrometry.^62^

The adverse in-utero and early life environment, exposures, and nutrition can impact the epigenome of the fetus and newborn.^63^ For example, intraamniotic infection results in systemic fetal inflammatory response characterized by cytokine storm that affects the DNA methylation pattern in the fetus.^64,65^ Maternal exposure to illicit drugs like opioids, cocaine, and cannabis can cause epigenetic changes in the fetus.^66,67^ Infants born prematurely are exposed to life saving measures with yet unknown long term effects such as excessive oxygen and corticosteroids that may have an effect on their epigenome.^68^ The epigenetic changes secondary to periconceptual, in-utero and early-life environmental stressors of the fetal and newborn period should be factored in the interpretation of the integrated multiomics data and their clinical relevance while taking an genome-first approach to precision medicine.

An ongoing prospective population-based birth cohort study in six European countries recruited 31,472 mother-child dyads and selected a sub-cohort of 1033 mother-child dyads for the initial exposome-wide association study. Eighty-five prenatal and 125 postnatal exposures and the child’s lung function at 6–12 years of age were evaluated.^69,70^ The study found that prenatal exposure to perfluorononanoate and perfluorooctanoate was associated with abnormal lung function. Postnatal exposure to copper, ethyl-paraben, phthalate metabolites, house crowding, and facility density around schools were associated with abnormal lung function. Another large prospective cohort study is underway in Europe to characterize the human exposomes in working life.^71^ The Exposome Project for Health and Occupational Research aims to develop detection and analytical platforms with high-throughput technologies to enable future research in this domain.

## Integrated multiomics

**Fig. 2:**
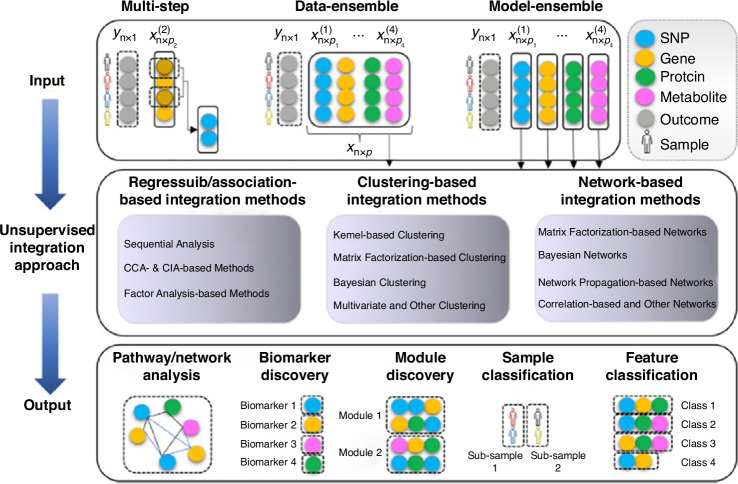
Approaches to unsupervised multiomics data integration. 1. Multi-step approach utilizes one of the three regression/association-based statistical methods for integration–sequence analysis, canonical correlation analysis/ co-inertia analysis, or factor analysis. 2. The data ensemble approach creates unified multiomics input data using one of the four clustering-based statistical integration methods (kernel-based, matrix factorization-based, Bayesian, or multivariate clustering). 3. The model ensemble approach utilizes one of the four network-based statistical methods (matrix factorization, Bayesian, Network propagation, or correlation-based) to analyze each multiomics data separately and combine the results to discover novel biomarkers, functionally similar modules, and classify samples. Reproduced from open access source Vahabi, N., & Michailidis, G. (2022). Unsupervised Multiomics Data Integration Methods: A Comprehensive Review.

Integrative multiomics, the combination of multiple omics data layered over each other, including the interconnections and interactions between them, helps us understand human health and disease better than any of them separately.^72,73^ Several approaches and computational platforms are available to achieve this integration. The integration process can be classified in several ways. One type of categorization of the integrative approach is early (concatenation-based), mixed (transformation based), intermediate, late (model-based), and hierarchical.^74^ Another broad way of classifying the integration platforms is supervised and unsupervised based on whether machine learning models are trained using data labels.^75^ Multiomics integration methodologies can be classified into the following approaches: 1. Regression/association-based methods, 2. Clustering-based methods, and 3. Network-based methods. Each approach uses one of the three methodologies, namely a. multistep and sequential analysis, b. data-ensemble, and c. model-ensemble. (Fig. 2) In the data ensemble method, the different multiomics data layers are linked and combined to form a single input for analysis. In contrast, in the model ensemble method, each omics data layer is analyzed separately and fused for the integrated analysis. The bioinformatics tools available to achieve integration are ever-expanding, and the choice depends on the analysis’s objective. These complex tools are extensively used in pediatric oncology and other pediatric diseases. A multicenter prospective cohort study (*n* = 221) of respiratory syncytial virus bronchiolitis used affinity matrix, similarity network fusion, and spectral clustering method to integrate multiomics data (clinical, virus, nasopharyngeal microbiome, transcriptome, and metabolome data) and found four distinct clinically relevant disease endotypes.^76^ A Canadian study based on a South Asian birth cohort (*n* = 50) found novel biomarkers of early-onset childhood obesity by integrating gut microbiome (55 bacterial amplicon sequencing variants) and serum metabolome (73 serum metabolites) profiles using a supervised machine learning tool called Data Integration Analysis for Biomarker discovery using Latent cOmponent (DIABLO).^77^

## EHR data integration with multiomics

Integrated multiomics data coupled with clinical context is essential to accomplish the aim of precision medicine. Electronic Health Records (EHR) contain structured data such as administrative information, laboratory parameters, vital signs, anthropometric measurements, medications, and diagnosis codes.^78^ The clinical notes from various healthcare providers (primary and consulting physicians, registered nurses, respiratory therapists, pharmacists, dieticians, and social workers) and medical imaging constitute the unstructured data. The combined data from the EHR can help provide the clinical context for interpreting multiomics, and hence, integrating EHR with multiomics is crucial for creating clinically relevant and actionable data. The first step in this process, combining structured and unstructured data, could be accomplished using technologies such as Health Level 7 Fast Healthcare Interoperability Resources (HL7 FHIR).^79^ The next step is integrating curated EHR data with multiomics utilizing AI-powered Big Data analytics. To accomplish this, we need high-quality data from EHR that incorporates patient heterogeneity and diversity to allow machine learning (ML) tools to reproduce reliable output. One way to achieve this is by national or international multi-center studies, but patient privacy and data security issues are potential barriers. Newer ML platforms, such as Federated Learning allow institutions to train their models locally with patient data. Then, the institutions share trained models without patient data.^80^ The models shared by various institutions are aggregated to create a robust consensus model.^81^ Application programming interface (API) and related Substitutable Medical Apps, Reusable Technologies (SMART®) platform are showing promise in this process of integration.^82^ A bioinformatics study demonstrated that an integrated SMART clinic-genomics app can be created by developing separate SMART on FHIR clinical and genomic data adapters to directly payload data from the EHR and sequencer.^83^ Examples of clinic-genomic apps include Precision Medicine Genes +AI and SMART Precision Cancer Medicine (PCM).^84^ PCM is an EHR-agnostic open-source software that helps clinicians visualize patient-specific genomic data in the context of a population-level spectrum within their clinical workflow.

## Databases

The first step in harnessing the potential of integrative multiomics is creating a scalable, collaborative, secure digital environment that can be used to identify, collect, share, and retrieve genomic and multiomics data. Such digital infrastructures are essential for making the best use of large datasets to help deliver customized healthcare at a population level. Table 2 lists a few examples of such genomic data infrastructure development initiatives.

**Table 2:** Genomic Data Infrastructure Development Initiatives.

No.	Name	Description	Collaborators
1.	The Science and Technology Research Infrastructure for Discovery, Experimentation, and Sustainability (STRIDES) Initiative	A cloud-based data-sharing undertaking by NIH to help biomedical researchers	Google Cloud, Amazon Web Services, and Microsoft Azure
2.	NIH Cloud Platform Interoperability (NCPI) program	A federated genomic data ecosystem in which the researchers can discover and merge data from different platforms, such as Analysis Visualization and Informatics Lab-space (AnVIL), BioData Catalyst, Cancer Research Data Commons (CRDC), Gabriella Miller Kids First Pediatric Research Program, Database of Genotypes and Phenotypes (dbGaP), Sequence Read Archive (SRA)	NHGRI, NHLBI, NCI, NIH Common Fund, NCBI and NIH
3.	European Genomic Data Infrastructure (GDI) program	Transnational access to genomic, related phenotypic, and clinical data to support the 1+ Million Genomes initiative	20 member states of the European Union

*NHGRI* National Human Genome Research Institute, *NHLBI* National Heart, Lung, and Blood Institute, *NCI* National Cancer Institute, *NCBI* National Center for Biotechnology Information, *NIH* National Institutes of Health.

## Data mining and bioinformatics

Bioinformatics, the science of using computational methods to analyze and understand biological data, utilizes data mining and knowledge discovery in databases, two interlinked processes for unearthing trends and patterns in large genomic and multiomics databases to generate novel clinically valuable insights and applications.^85^ Data mining relies on machine learning and advanced statistical methods to recognize patterns in clinical big data.^86^

The investments made in developing large bioinformatics datasets worldwide have provided a tremendous opportunity for data mining. Some public genomic databases that could be used for data mining were mentioned earlier. Others include Gene Expression Omnibus (GEO), Cancer Genome Atlas, International Cancer Genome Consortium, Comparative Toxicogenomic Database, and Biologic Specimen and Data Repositories Information Coordinating Center. The data mining process involves three main steps—identifying an appropriate model (predictive or descriptive), choosing a task to implement the model (classification, estimation, prediction, association, clustering, and descriptive visualization), and using analytical methods to complete the process.

Let us outline an example to help understand the opportunities and challenges in bioinformatics data mining. GEO is a public repository containing functional genomics datasets by the global research community, maintained by the US National Center for Biotechnology Information. The original data supplied by the researchers are curated into study-level records called GEO datasets and gene-level records called GEO profiles that can be queried and analyzed by other researchers using internet-based data mining tools.^87^ A GEO dataset-based study used gene set enrichment analysis to find the relevance of MCYN-associated genes in predicting the prognosis of pediatric neuroblastoma.^88^ Although genomic data mining opens up many research avenues, the data in the public repositories are underutilized, especially in pediatrics. The challenge researchers face in multiomics data reuse from these huge repositories is the technical complexity of accessing and analyzing data. Several user-friendly software applications have been developed to overcome this problem. Examples of such applications include DataMed – an open-source biomedical data discovery system; GEO RNA-seq Experiments Interactive Navigator(GREIN) - an interactive web platform for re-analyzing GEO RNA-seq data; OMICtools- directory of more than 4400 web-accessible tools related to genomics, transcriptomics, proteomics and metabolomics, Datasets2Tools - search engine for bioinformatics datasets, tools and canned analyses.^89–92^

## Clinical and research applications

### Clinical applications

We will review some of the advances in integrative multiomics and genomics that have been clinically studied that promise to deliver precision medicine to the bedside in specific areas of pediatric oncology, pediatric critical care, and pediatric diabetes. Tissue-specific transcriptome analysis or RNA-sequencing has been increasingly utilized in research studies for resolving undiagnosed diseases with diagnostic yield ranging from 7 to 35%.^93^ More recently, it is being implemented in clinical laboratory reporting for the resolution of non-coding and splice variants detected by genome sequencing. The use of RNA-seq with genome sequencing promises to enhance the interpretation of such genetic variants for improved clinical care.

A study utilized a multiscale RNA clustering approach to define pediatric cancers at a molecular level and applied the method to a retrospective dataset of 13313 transcriptomes to classify pediatric cancers. The new diagnostic classification was used to design a deep learning model and validated in a prospective cohort with 85% accuracy.^94^ A population-based pediatric neuro-oncology study aimed to identify the molecular neuropathology of pediatric CNS tumors.^95^ The authors developed a neuro-oncology-specific NGS gene panel and a DNA methylation-based classification system to achieve this. Both the omics data were integrated with the histopathology reports. Multiomics integration improved diagnostic accuracy, aided detection of mutations relevant to diagnosis and treatment, identified precancerous syndromes, and predicted prognosis better. A study aimed to evaluate the combination of exome and transcriptome sequencing and SNP array to provide precision cancer therapy for children and young adults (*n* = 59) with relapsed and refractory non-CNS solid tumors.^96^ The investigators found that 51% of the study participants had clinically actionable mutations, including germline and somatic events (including a single nucleotide variant, an amplification, a deletion, an indel, or a fusion gene), that could be targeted in the ongoing clinical trials.

An Australian cohort study included 290 critically ill infants and children referred from neonatal intensive care units (47%), pediatric intensive care units (39%), and other hospital units nationwide to the Acute Care Genomics program comprising a national panel of experts.^97^ The trio analysis (patient and both parents) was done in 94% of the cohort. Ultra-rapid genome sequencing was the initial testing offered, followed by transcriptomics and proteomics analysis if they remained undiagnosed after genome sequencing alone. The integrative multiomics approach increased the diagnostic yield from 47% to 54%, with an impactful alteration in the management plan in approximately 1/3rd of the cohort. A systematic review including 21 prospective studies (*n* = 1654) aimed to synthesize the clinical utility of exome and genome sequencing in critically ill infants younger than 1 year. The study categorized the utility of genomics results as treatment change, redirection of care, prognostic information, reproductive information, and screening or subspecialty referral. The review found that a significant percentage of critically ill infants (mean—37% [13–61%]) undergoing genomic evaluation experienced clinical utility.

Armenteros et al. reported the molecular signatures in newly diagnosed type 1 diabetes patients (*n* = 97) from the pan-European consortium that predicted the rate of beta cell loss after the diagnosis using multiomics (integrated transcriptomic, genomic, targeted proteomic, lipidomic, metabolomic, and immunomic data) factor analysis.^98^ The two specific molecular signatures predictive of rapidly declining beta cell functional loss were identified. One of them was associated with immune pathways, and the other correlated with viral infection. The study showed the promise of using multiomics to identify newly diagnosed type 1 diabetic patients at risk of rapid disease progression and design multiomics-informed clinical trials, enabling the development of precision therapeutics.

The selected studies discussed above showed the usefulness of integrated multiomics in the diagnosis and management of pediatric cancers, critical illness, and diabetes. However, the relevance of the availability of these technologies, their utility, and applicability in clinical care spans various spheres of pediatric healthcare.

### Research applications

Traditionally, genomics research hypotheses have been postulated with the phenotype-first approach. The reasons for the phenotype-first approach in genomics research include readily available clinical data from EHR, easily traceable family records, information regarding exposomes that can be inferred from routinely collected information such as zip codes, and infrastructures for data sharing that already exists or at least easier to establish.^99^ However, this approach creates challenges and limits the yield of clinically helpful knowledge from the research endeavors. The EHR records are incomplete and not uniform across institutions, zip code/ insurance-based estimation of exposomes, including psychosocial stress, provides cross-sectional data and does not consider the lifetime experience of the participants.^100,101^ There are difficulties in scaling the datasets on a global stage because of the differences in the healthcare delivery models in LMIC countries and ascertainment bias limiting our ability to delineate penetrance, expressivity, and variant pathogenicity.^100,101^ Thus, the phenotype-first approach has drawbacks and is not ideal for research or for advancing precision medicine in children.

The genotype-first approach in genomic and multiomics research can overcome the limitations of the phenotype ascertainment and clinical informatics approaches and enhance the subclassification of diseases by widening the phenotypic spectrum and reverse phenotyping.^101,102^ Reverse phenotyping, where the genotypes of the research participants are known at recruitment, and a hypothesis is formulated to correlate with new phenotypic data, forms the basis for genomic ascertainment research. Genomic ascertainment research can help us uncover unrecognized or undiagnosed phenotypes, find an association between a new genotype and disease, and conduct an ex vivo phenotype analysis. The National Human Genome Research Institute (NHGRI) Reverse Phenotyping Core has piloted this research approach, mostly in adults utilizing the ClinSeq cohort, highlighting the feasibility of this approach.^103,104^ The tenets of the reverse phenotyping research strategy include an informed consent process that allows recontact and data sharing, sustaining the participation of the research subjects, institutions, and stakeholders, clarity and transparency in sharing results with participants, and networking to recruit large cohorts. A framework and challenges of conducting reverse phenotyping research and their possible solutions are presented in Table 3.^101^

**Table 3:** A. A Framework for a Genotype-First Approach B. Challenges in Genotype-First or Reverse Phenotyping Approach in Pediatrics.

A
A. Create a strategic plan at the outset and secure written agreement for pledged commitments from stakeholders
B. Include broad data sharing and the ability to recontact participants in the genomic research informed consent process
C. Establish long-term, trusting relationships with study participants: success depends on the participants’ willingness to return for studies
D. Generate and maintain institutional engagement and support for follow-up studies: establishing and maintaining a reverse phenotyping resource requires institutional commitment and material resources of staff, money, and time.
E. Define what results will be returned, to whom, and by whom: lack of clarity around which study participants will receive results and who is responsible for delivery of these results can diminish participants’ trust and risk their participation in future studies.
F. Invest in adding new cohorts through networking with other investigators: there is power in numbers, especially for rare variants, so it is crucial to have a large pool of participants from which to draw.

B		
No.	Challenges	Potential Solution
1.	Need for long-term follow-up of the pediatric patients and their families	Include a follow-up provision in all informed consent forms for research with a clear definition of the purpose
2.	Need for storing genomic data in open-science databases	Strengthen the data infrastructure to prevent misuse and help parents make an informed choice about data sharing in the research community
3.	Cost burden of long-term follow-up and data storage	Communicate the impact and return for investment to stakeholders in tangible terms
4.	Ethical conundrum in sharing results of genomic research with patients and their families	Investigators should develop a strong plan for returning research findings during the design phase and should be mandated by the institutional review boards before study approval
5.	Recruiting large prospective cohorts of representative diversity	Foster global networks and community partnerships

Adapted from “Genotype first: Clinical genomics research through a reverse phenotyping approach” by Wilczewski et al. Cell Press Open Access.

A study aimed to find the relationship of gene-gene interactions (epistasis) in the pathophysiology of ASD used the genotype-first approach and found that single nucleotide polymorphisms in the Ras/MAPK pathway contribute to idiopathic ASD.^105^ The study identified that dysregulation of the GPR141 gene may have a role in idiopathic ASD. A study of steroid-resistant nephrotic syndrome utilized exome sequencing and a reversed phenotyping strategy to identify novel clinical signs and prognostic factors associated with genetic nephropathies that could be differentiated from podocytopathies.^106^ Thus, a genome-first approach overcomes the drawbacks of a phenotype-first approach and can identify multiple phenotypes for the same gene defect, quantify the gene-dose effect, and help in prognostication.

## Road map to AI-based integrative multiomics approach for precision medicine in pediatric healthcare and research

We present a road map for implementing integrative multiomics in pediatric clinical care. (Fig. 1) Large prospective pediatric cohorts with representative diversity should be developed with global public and private partnerships with secure and scalable data infrastructure. Multiomics data from such a cohort of patients should be integrated using artificial intelligence/machine learning tools described earlier in the review. Such integrated multiomics data should be combined with curated EHR data to create databases and data warehouses with an open-science model. Clinicians and researchers should be equipped with user-friendly, accessible bioinformatics tools to access the data warehouses with a genomics-first approach. This method helps clinicians precisely subtype patients with complex diseases with overlapping phenotypes, and researchers discover accurate biomarkers and targeted therapeutics. Such a road map would advance patient-centered, value-based precision healthcare in pediatrics.

## Precision medicine—current challenges and the future

The challenges facing implementing precision medicine in routine clinical practice can relate to 1. Data, 2. Cost 3. Bioethics, and 4. Legal issues. We need huge scalable, and interoperable data for the AI/ML models to prove they can make accurate predictions for widespread implementation in clinical care.^4^ We need to establish accessible digital infrastructures to collect, store, and share data of large magnitudes in compliance with FAIR guidelines.^107^ We need data that would inform us about the human genome’s regulatory/non-coding component, which will complete the puzzle and enhance our understanding.^108^ We need a diverse population in our datasets so that the ML models are not biased in their prediction.^109^ We must develop capabilities for out-of-sample cross-validation of the AI/ML models to produce generalizable data. AI models must recognize heterogeneity in the disease mechanisms from population data having the same phenotypic labels. Data intended for precision medicine must capture patient-centered outcome measures, and the predictions should be tuned to factor in those outcomes while predicting treatment responses. Implementing multiomics data collection and integration with the EHR at a population level would incur excessive costs. The analytic tools needed to handle integrated multiomics-EHR big data cannot be made accessible without the will and investment of policymakers. Healthcare systems dependent on medical insurance reimbursement need the payers’ acceptance and support for the practice of precision medicine. Existing reimbursement models will be insufficient to sustain the widespread implementation of precision medicine.^110^ The biggest ethical challenge facing the implementation of precision medicine is the balance that needs to be struck between inclusivity and diversity in the big datasets and the protection from potential harms from breaches in data safety and privacy, implications in future insurance premiums, discrimination in society, and employment. On the legal and regulatory front in the US, the genomics and multiomics tests are categorized as laboratory-developed tests (LDT).^111,112^ LDTs are currently not regulated by the Food and Drug Administration. So, LDTs can be marketed without proof of clinical validity and utility.

The first step in realizing the hope of precision medicine is delineating the existing infrastructure and barriers to advancing precision medicine at a local, regional, and global level.^113^ The second step is to create a collaborative network of various stakeholders - physicians, other healthcare providers, researchers, public and private research funding agencies, healthcare systems, academic institutions, healthcare payers, industry partners from genomics and multiomics diagnostic, pharmaceuticals and biological, bioinformatics, governmental regulatory agencies, and public health policymakers—with a shared vision of precision medicine. Such a network must include patients, their families, and community support groups. The third step is to invest in the education and training of the workforce, including clinical geneticists, computational genomics, and data scientists, to implement precision medicine sustainably.^114^ The current generation of healthcare providers has to be provided with the necessary knowledge and skills to adopt and incorporate the practice of precision medicine into traditional practice. A diverse precision medicine workforce should be developed in healthcare, medical research, data science, bioinformatics, computational, and systems biology for the future demands of the field. Patients have to be educated about the benefits of precision medicine and encouraged to participate and contribute to the diversity of the data. Fourth, an informed consent process should be streamlined to enable patients from diverse socioeconomic strata to participate in genomics research. Fifth, multiomics and stored EHR data should be regulated by law to protect patient privacy, prevent discrimination, and make data access and reuse easier. Health Insurance Portability and Accountability Act (HIPAA) laws ensure genomic data in EHRs are protected from unauthorized access, requiring EHR systems to follow strict regulations for the secure handling, storage, and transmission of genetic data to maintain patient privacy. The Genetic Information Nondiscrimination Act of 2008 (GINA) prohibits discrimination based on genetic information in health insurance and employment. However, its protections are limited and do not extend to other types of insurance, such as long-term disability coverage or life insurance. These patient safeguards have to be strengthened further. Sixth, a combination of government-initiated “top-down” and healthcare systems-initiated “bottom-up” approaches should be pursued in precision medicine research, innovation, and implementation.^115^ Seventh, for the successful implementation of precision medicine, the insurance reimbursement model must change from fee-for-service to value-based healthcare.^116^ The health systems assessment framework should become patient-centered and reformed by identifying and obtaining consensus from various stakeholders.^117^

Innovations in genomics, multiomics, and AI have begun to create a paradigm shift in our understanding of human biology. The patient-centered precision approach to diagnosing and treating certain diseases like cancer has shown us a glimpse of the future landscape of healthcare. Research aimed at translating the successes of precision oncology into other common public health problems like neuropsychiatric and cardiometabolic disorders, has informed us of the potential to implement precision medicine at a population level. Stakeholders’ confidence in the promise of precision medicine has enabled national and global collaborations on a scale unseen in the past to establish the infrastructure needed for the sustained innovation and implementation of precision medicine. Adopting the principles of precision medicine in the research of disorders affecting newborn infants, children, and adolescents has shown us how to bring about patient-centered, value-based transformation in the practice of pediatrics and its subspecialties. We are far from implementing precision medicine in our day-to-day practice. However, we know the pathway to be taken to get there. We envision datasets similar to the scale of All of Us that would be created to include children with built-in ethical protections. Common pediatric public health burdens such as neurodevelopmental disorders, asthma, obesity, and prematurity would be subtyped based on distinct etiopathogenic mechanisms, diagnosed early by novel, accurate biomarkers, treated effectively with targeted therapeutics, prognosticated better by leveraging integrated multiomics linked with EHR and AI.
